# Comparative Study of Indirect Fluorescent Antibody, ELISA, and Immunochromatography Tests for Serological Diagnosis of Bovine Babesiosis Caused by *Babesia bovis*

**DOI:** 10.3390/ani11123358

**Published:** 2021-11-24

**Authors:** José Juan Lira-Amaya, Grecia Martínez-García, R. Montserrat Santamaria-Espinosa, Roberto O. Castañeda-Arriola, Juan J. Ojeda-Carrasco, Guillermina Ávila-Ramírez, Julio V. Figueroa-Millán

**Affiliations:** 1 Babesia Laboratory Unit, CENID-Salud Animal e Inocuidad, INIFAP, Carr. Fed. Cuernavaca-Cuautla No. 8534, Col. Progreso, Jiutepec 62550, Mexico; lira.juan@inifap.gob.mx (J.J.L.-A.); martinez.grecia@inifap.gob.mx (G.M.-G.); santamaria.rebeca@inifap.gob.mx (R.M.S.-E.); 2 La Posta Experimental Field, INIFAP, Carr. Fed. Veracruz-Cordoba Km. 22.5, Paso del Toro, Medellin 94277, Mexico; castaneda.roberto@inifap.gob.mx; 3 UAEM University Center Amecameca, Autonomous University Mexico State, Carr. Amecameca-Ayapango Km. 2.5, Amecameca 56900, Mexico; jjojedac@uaemex.mx; 4 Faculty of Medicine, National Autonomous University of Mexico, Circuito Escolar 411A, Copilco Universidad, Ciudad de México 04510, Mexico; guilleavila2000@yahoo.com

**Keywords:** bovine babesiosis, serological diagnosis, ELISA, ICT, IFAT

## Abstract

**Simple Summary:**

Currently serological diagnosis of bovine babesiosis is based on the detection of *Babesia*-specific antibodies (immunoglobulin-G). Antibody detection is commonly used in seroepidemiological studies or in the assessment of antibabesial antibody titers after cattle vaccination. The indirect fluorescent antibody test (IFAT) and enzyme-linked immunosorbent assay (ELISA) are the most widely used diagnostic tests, although there their implementation has some drawbacks, principally due to the requirements for trained personnel, specific materials, and special laboratory equipment. This study compared a newly designed rapid immunochromatography test (ICT), which has been reported recently and used for *Babesia bovis*-specific antibody detection with promising results, with an in-house ELISA for the serological diagnosis of cattle exposed to *B. bovis* (*Babesia bovis*) in Mexico. Higher sensitivity and specificity values were found by ICT, proving its effectiveness over ELISA. ICT also had better concordance than ELISA when IFAT was used as the “gold standard”. The rapid ICT was shown to have diagnostic utility for the detection of antibodies against *B. bovis* and could be used as a field test in Mexico due to its practicality, as it does not need laboratory equipment for implementation and interpretation of results.

**Abstract:**

The indirect fluorescent antibody test (IFAT) is the most frequently used test to conduct seroepidemiological studies so far, and it is regarded as the "gold standard" test for the serological diagnosis of bovine babesiosis. The aim of the present study was to compare the enzyme-linked immunosorbent assay (ELISA) and the rapid immunochromatography test (ICT) for use in the serological diagnosis of cattle exposed to *B. bovis* in Mexico. The evaluation of test performance was carried out with 30 positive and 30 negative reference sera. A total of 72 bovine sera samples collected from cattle in a region with endemic bovine babesiosis were analyzed by ELISA and ICT, and the results were compared with those of IFAT. Kappa value (k) was also calculated to determine the agreement between tests. The sensitivity and specificity of ELISA for detecting antibodies against *B. bovis* were 87% (26/30) and 80% (24/30), respectively. The sensitivity and specificity of ICT for detecting antibodies against *B. bovis* were 90% (27/30) and 83.3% (25/30), respectively. The overall concordance determined for ELISA and ICT was 94.4% (68/72) and 98.6% (71/72), respectively, when the results were compared with those of IFAT. ICT was more sensitive and specific in this comparative study, showing good strength of agreement (k = 0.79) with respect to IFAT. ICT combines a strip-based assay system that is fast, practical, and sensitive for detection of antibodies to *B. bovis*, which suggests that it could be applied in the field without requiring any laboratory equipment for its use and interpretation of test results.

## 1. Introduction

Livestock production in Mexico is considered one of the main activities of the agri-food sector, ranking second only after crop production. The predominant system for meat and milk production in tropical and subtropical regions is extensive grazing [1]. Currently, the total cattle population in Mexico is estimated at 35.2 million head, contributing to the production of approximately 2 million tonnes of meat and 12.5 million liters of milk per year [2]. The occurrence of certain infectious diseases in cattle, including tick-borne diseases such as babesiosis, is a problem of great economic importance to the livestock industry [3].

Bovine babesiosis, especially caused by *Babesia bovis* and *Babesia bigemina*, is a tick-borne hemoprotozoan disease that is widely distributed in tropical and subtropical regions all over the world. The infection is clinically characterized by fever, listlessness, hemoglobinuria, hemolytic anemia, and death in acute untreated cases [4]. In Mexico, the main vector of both *Babesia* species is the tick *R. microplus* [5]. Bovine babesiosis represents a limitation to development and productivity in tropical and subtropical livestock production regions all over the world [6]. The economic losses may be on the order of USD 10 billion per year worldwide [7], associated with low milk production and decline in daily weight gain of infected animals, along with the high costs of treatment and the application of control measures for tick vectors [8]. Currently, 75% of the cattle population raised in regions with a high incidence of *R. microplus* ticks in Mexico is at risk of becoming infected with *B. bovis* and *B. bigemina* [8,9].

Routine laboratory diagnosis consists of identifying intraerythrocytic *Babesia* sp. forms during microscopic examination of Giemsa-stained blood smears [5]. Serological tests are commonly used to detect *Babesia*-specific antibodies in serum samples, such as the indirect immunofluorescence assay (IFAT) and enzyme-linked immunosorbent assay (ELISA) [10]. The IFAT is the most frequently used test in seroepidemiological studies and so far is regarded as the “gold standard” test for serological diagnosis of bovine babesiosis [11]. However, both serological tests have several limitations, as their implementation requires trained personnel and special laboratory material and equipment [12]. Recently, rapid immunochromatography tests (ICTs) have been reported to be effective when used for the detection of specific antibodies against *B. bovis* or *B. bigemina* in cattle [13,14,15,16,17]. The ICT is a rapid, membrane-based lateral flow immunoassay that does not require any laboratory equipment for result analysis and has been reported to have high diagnostic sensitivity. In addition, it has the great advantage that it can be used in clinical and field conditions directly on farms [12].

The aim of the present study was to compare the enzyme-linked immunosorbent assay (ELISA) and the rapid immunochromatography test (ICT) for use in serological diagnosis of cattle exposed to *B. bovis* in Mexico.

## 2. Materials and Methods

### 2.1. Sample Size Calculation

The sample size of the cattle population was determined according to the mathematical formula described for research studies [18] using the Raosoft^®^ program (freely available online: http://www.raosoft.com/samplesize.html accessed on 1 July 2020). The formula n = [N (Z^2^) × p (1 − p)]/[d^2^ (N − 1) + (Z^2^) p (1 − p)] was applied, where n is the required sample size, N is the population size, Z is the confidence value (95%), p is the approximate prevalence, and d is the absolute accuracy level (5%). The approximate prevalence for the sampled area was 80%, as previously reported in a study performed in Chiapas State, Mexico [19].

### 2.2. Serum Samples

#### 2.2.1. Reference Serum Samples

Positive and negative serum samples classified by IFAT were used to perform the evaluation using ELISA, ICT, and IFAT. Thirty *Babesia* sp.-negative serum samples were collected from cattle born and raised in Amecameca municipality, State of Mexico, Mexico (2420 m above sea level, sub-humid temperate climate), considered a naturally *Rhipicephalus (Boophilus)* tick-free area and, therefore, a *Babesia*-free area [20,21]. Thirty *B. bovis*-positive serum samples from dual-purpose cattle in an area with endemic bovine babesiosis in Paso del Toro, Veracruz, Mexico (10 m above sea level, tropical climate conditions) [5]. The reference serum samples were used to assess the performance of ELISA, ICT, and IFAT.

#### 2.2.2. Field Serum Samples

Blood samples from 72 cattle were collected at a farm located in a babesiosis endemic area (Pichucalco, Chiapas, Mexico). The serum was separated immediately by centrifugation, and then stored in aliquots at −20 °C until use. The serum samples were used to estimate the degree of agreement between ELISA and ICT and compare those against IFAT.

### 2.3. Pre-Adsorption of Serum Samples 

To avoid nonspecific antibody binding in the tests, all serum samples were subjected to a pre-adsorption process with *E. coli* cell lysate, as described previously [22,23]. Briefly, 200 µL of *E. coli* TOP10 (uninduced) cells cultured in LB medium and stored at −80 °C were resuspended in 1 mL of phosphate-buffered saline (PBS) containing 500 µL acid-washed glass beads (Sigma-Aldrich, St. Louis, MO, USA). Then, the suspension was homogenized using a mechanical shaker for 30 s at maximum speed and immediately placed on ice for 30 s; these steps were repeated until 8 cycles were completed. Subsequently, the lysate suspension was centrifuged at 18,620× *g* for 8 min at room temperature and the supernatant was separated from the pellet for use in the assay. The ELISA microplates were coated with 50 µL (100 µg/mL) of *E. coli* lysate suspension and prepared as described in Section 2.4. The serum samples were added to the ELISA microplates and incubated for 30 min at 37 °C, then transferred to another ELISA microplate, which has been previously coated with the lysate, and the microplates were incubated again for 30 min at 37 °C. The absorbed serum samples were recovered and stored in aliquots at −20 °C until analysis.

### 2.4. Preparation of Recombinant Protein MSA-1 and Sheep Anti-rMSA-1 IgG

*Babesia bovis* recombinant merozoite surface antigen 1 (rMSA-1) was expressed and prepared as previously described [24]. Briefly, transformed *E. coli* TOP10 cells containing the recombinant pBAD/ThioTOPO®, (ThermoFisher Scientific, Waltham, MA, USA) plasmid inserted with the *msa-1* gene were resuspended in lysis solution containing sarcosyl (0.03%) and protease inhibitors DNase and RNase and incubated for 3 h at room temperature with gentle agitation. The recombinant *B. bovis* MSA-1 protein was recovered using a Pro-Bond Purification System on nickel-charged Ni-NTA affinity resin (ThermoFisher Scientific, Waltham, MA, USA) according to the manufacturer instructions, and purification quality was analyzed by electrophoresis in 12% polyacrylamide gels [10,24]. Finally, the rMSA-1 was dialyzed against PBS (pH 7.4) and concentrated using Amicon 50K MWCO filters (Corning, Tewksbury, MA, USA). Four male Pelibuey sheep (˃12 months of age) were immunized with 500 µg of rMSA-1 mixed with an equal volume (*v/v*) of Montanide ISA 70VG adjuvant (SEPPIC, Fairfield, NJ, USA) by administration of 5 subcutaneous injections into the scapular area at 14-day intervals. Total IgG was purified from sheep serum with Protein G Sepharose (Sigma-Aldrich) according to the manufacturer’s instructions. The concentration and dialysis procedures for sheep anti-rMSA-1 IgG were the same as those performed for rMSA-1.

### 2.5. Enzyme-Linked Immunosorbent Assay (ELISA)

The test was performed similarly to the established protocol described previously [25] with slight modifications. For this test, 96-well ELISA microplates (Corning, Tewksbury, MA, USA) were coated with 50 µL of rMSA-1 (1.8 µg/mL) diluted in carbonate/bicarbonate buffer (pH 9.6) (Sigma-Aldrich) and incubated at 4 °C overnight. The ELISA microplates were blocked with 50 µL/well of 5% skim milk in PBS-0.1% Tween 20. Triplicates containing 50 µL/well of the pre-adsorbed serum samples (1:200 dilution in PBS) were added and microplates were incubated for 1 hour at 37 °C. Then, 50 µL of rabbit anti-IgG bovine conjugated with horseradish peroxidase (1:10,000 dilution in PBS) was added to each well and incubated for 30 min at 37 °C. Tetramethylbenzidine substrate (Sigma-Aldrich) was then added to each well of the ELISA microplates (50 µL/well). After 30 min at 37 °C incubation, the absorbance value was determined using an iMARK microplate reader (Bio-Rad, Hercules, CA, USA) with a 650 nm filter. During the procedure, the microplates were washed 3 times between each step with PBS 0.1% Tween 20, except after the substrate was added. The results, obtained as optical density (OD) absorbance values for each serum sample, were expressed as a positivity index (PI) according to the following formula: PI = average OD each serum/average OD negative control + 3 standard deviations; serum with a PI value ≥ 1 was considered as positive and PI < 1 as negative for antibodies against *B. bovis*.

### 2.6. Immunochromatographic Test (ICT)

The bioconjugate was prepared by gently mixing *B. bovis* rMSA-1 (90 µg/mL) with 50 nm gold colloid nanoparticles (Ted Pella Inc., Redding, CA, USA) at a 1:10 volume ratio and then incubated for 2 h at room temperature with agitation (100 rpm). Subsequently, 1 mL of 20 mM borate buffer at pH 7.4 containing 1% bovine serum albumin (BSA) was added to stabilize and block the bioconjugate nanoparticles. After centrifugation for 30 min at 18,620 ×*g*, the supernatant was discarded and the pellet was resuspended in 1 mL of 20 mM borate buffer at pH 7.4 containing 1% BSA. The suspension was centrifuged again for 30 min at 18,620 ×*g* and the resulting pellet was resuspended in 200 µL of resuspension buffer for bioconjugate (1% (*w/v*) BSA, 0.02 M (*w*/*v*) Tris base, 0.003 M (*w/v*) sodium azide, and 20% (*w/v*) sucrose). rMSA-1 (1100 µg/mL) and sheep anti-rMSA-1 IgG (8700 µg/mL) were linearly jetted onto a test line and control line, respectively, of a nitrocellulose membrane with a plastic backing (Millipore, Burlington, MA, USA) using a XYZ3210 dispensing platform (BioDot, Irvine, CA, USA). Then, the membrane was dried for 30 min at 45 °C and cut in 4 mm wide strips using a CM5000 guillotine cutter (BioDot, Irvine, CA, USA)). The bioconjugate pads were manually impregnated with 4 µL of the bioconjugate and then dried for 30 min at 42 °C. Assembly of the strips was carried out as previously described [26], by attaching the nitrocellulose membrane, sample pad, bioconjugate pad (glass fiber), and absorbent pad slightly overlapping each part. The test was performed by adding 10 µL of pre-adsorbed serum on the sample pad, which was previously activated with a solution containing 0.05 M (*w/v*) Tris base, 0.5% (*w/v*) casein, 1% (*w/v*) polyvinylpyrrolidone (PVP), and 0.05% (*v/v*) Tween 20, followed by 100 µL of the running buffer solution (PBS at pH 7.6 with 1.7% (*w/v*) BSA and 3% (*v*/*v*) Tween 20). The result for detection of antibodies to *B. bovis* was visually interpreted 10 min after sample addition based on the appearance of one colored band at a control line (negative) and two colored bands (positive) at control and test lines. Any other result was considered an invalid test [27].

### 2.7. Indirect Fluorescent Antibody Test (IFAT)

Microscope slides of *B. bovis*-infected erythrocytes derived from in vitro culture were used as antigen [7]. The slides were dried in silica beads for 30 min at 37 °C and subsequently fixed with acetone for 10 min. Then 10 µL of the pre-adsorbed serum samples (1:80 dilution in PBS) was placed on the slides and incubated for 30 min at 37 °C, followed by 3 washes with PBS in gentle agitation for 5 min each. Then, 10 µL of goat anti-IgG bovine conjugated with fluorescein isothiocyanate (diluted 1:1600 in PBS) was added to each sample and washed as described above. Finally, the slides were mounted and visualized with a Leica DMLB epifluorescence microscope (Leica, Wetzlar, Germany) using a 100× objective.

### 2.8. Statistical Analysis

The performance of ELISA, ICT, and IFAT was evaluated in terms of sensitivity, specificity, positive predictive value (PPV), negative predictive value (NPV), positive likelihood ratio (+LR), negative likelihood ratio (−LR), and kappa value with 95% confidence interval (CI) using the VassarStats program (freely available online: http://vassarstats.net/ accessed on 1 February 2021). The strength of agreement with kappa values was interpreted as poor, <0.20; fair, 0.21 to 0.40; moderate, 0.41 to 0.60; good, 0.61 to 0.80; or very good, 0.81-1.00 [15,28]. The diagnostic utility of the tests was measured according to the +LR (˃1) and −LR (<0.1) values, and was considered as highly relevant, good, fair, or poor [29].

### 2.9. Ethical Statement

The cattle were immobilized (<5 min) in a cattle crusher to collect the blood samples. Animal handling was conducted in accordance with Mexican regulation NOM-062-ZOO-1999 regarding technical specifications for production, care, and use of laboratory animals.

## 3. Results

### 3.1. Performance Evaluation of ELISA and ICT 

**Figure 1:**
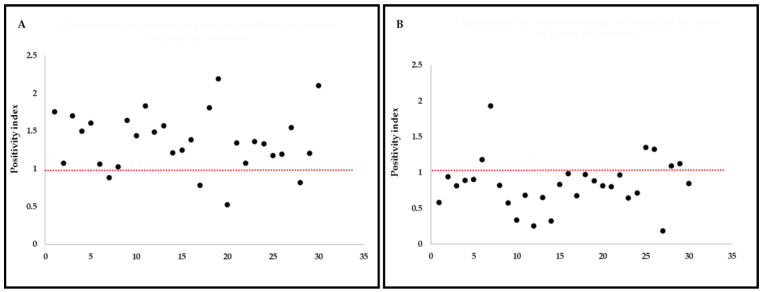
Scatter chart of values according to positivity index for each serum sample in ELISA: (**A**) 30 *B. bovis*-positive cattle serum samples and (**B**) 30 *Babesia* sp.-negative samples compared to IFAT; samples were considered positive by ELISA at a positivity index >1 and negative at <1, respectively.

**Figure 2:**
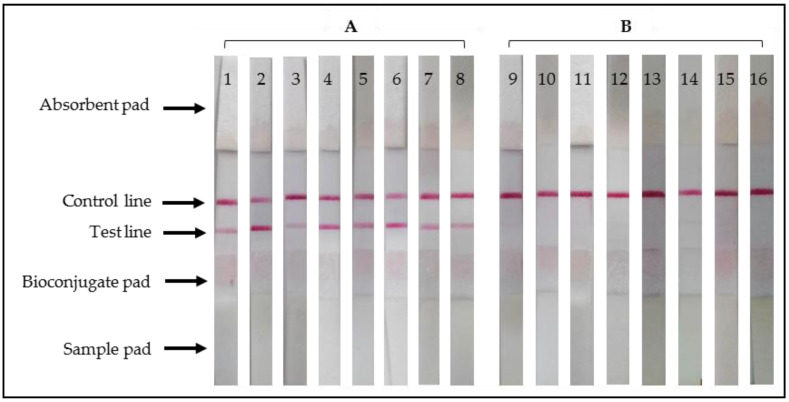
ICT strips used for detection of antibodies to *B. bovis*: examples after applying (**A**) positive and (**B**) negative reference bovine serum samples. Lanes 1–8, positive result for detection of antibodies to *B. bovis* (colored bands at control and test lines); lanes 9–16, negative result (colored band at control line).

The results obtained in the performance evaluation of tests are shown in Table 1. The sensitivity and specificity of ELISA for detection of antibodies to *B. bovis* (Figure 1) were 87% (95% CI: 68.3–95.6%) and 80% (95% CI: 60.8–91.5%) and the probability of getting a positive (PPV) or negative (NPV) result using the test was 81.2% and 85.7%, respectively. Similarly, the ICT for detection of antibodies to *B. bovis* (Figure 2) showed a sensitivity of 90% (95% CI: 72.3–97.3%) and a specificity of 83.3% (95% CI: 64.5–93.6%), with a probability of PPV and NPV at 84.3% and 89.2%, respectively. 

### 3.2. Comparison between ELISA and ICT for Detection of Antibodies to B. bovis

The test showing the highest number of positive results for detection of antibodies to *B. bovis* in field serum samples was the ICT one (Table 2). The ICT detected antibodies in 70 serum samples (97.2%), including one sample that was negative by IFAT. Three serum samples were negative by IFAT and ELISA, but four samples that were negative by ELISA were positive by IFAT (Table 3). When the results of ELISA and ICT were compared with those of IFAT, the overall concordance was 94.4% (68/72) and 98.6% (71/72), respectively. The sensitivity was 94.2% (65/69) and 100% (69/69), and the specificity was 100% (3/3) and 66.6% (2/3), respectively (Table 4). The strength of agreement with respect to IFAT was moderate for ELISA (k = 0.57) and good for ICT (k = 0.79). ELISA and ICT showed fair and good diagnostic utility, respectively, according to the interpretation of +RV and −RV values (Table 4). 

## 4. Discussion

Different tests have been described and used routinely for the detection of specific antibodies in cattle after exposure to *Babesia* sp. [7,30]. These tests are not useful for diagnosing bovine babesiosis during the clinical phase of the disease but are used for epidemiological studies or to evaluate antibabesial antibody titers after cattle vaccination [4,7,24,30,31]. The IFAT provides adequate sensitivity and is easy to perform [12]; however, it has some disadvantages in the analysis of samples, such as the low number that can be performed per day, depending on the operator’s ability and expertise, and the results are influenced by the subjective judgment of the analyst [32]. ELISA allows processing of a higher number of samples in a working day, and since it is an automated method, another advantage is the objectivity of the results interpretation [12]. Among the main disadvantages of ELISA is that the procedure involves several steps for execution, which makes it costly, and like IFAT, it requires special laboratory equipment for sample analysis and result interpretation [14]. Recently, the development of new and innovative diagnostic methods, such as an immunochromatography test (ICT) based on a lateral flow membrane, have changed the landscape for users, facilitating the detection of antibodies or soluble antigens [33,34]. These tests provide specific results in a timely manner and a short time, and they can be applied in laboratories, veterinary clinics, or directly on farms without the need for any equipment to detect or display the results of the assay [35].

In the current study, a comparison of ELISA and ICT with IFAT was performed to assess their feasibility for wider use in the serological diagnosis of cattle exposed to *B. bovis* in Mexico. The evaluation of ELISA performance showed that the sensitivity and specificity rates for detecting antibodies against *B. bovis* were 87 and 80%, respectively. The sensitivity and specificity of an indirect ELISA instrumented in Mexico and based on *B. bovis* recombinant merozoite surface antigen 1 (MSA-1) were 80 and 97%, respectively [36]. The ELISA in this study was performed as described previously [36]; however, some modifications were made, including the use of a different set of positive and negative control sera and a different rMSA-1 antigen batch, and replacement of commercial goat serum (anti-IgG bovine) conjugated with alkaline phosphatase (ALP) by commercial rabbit serum (anti-IgG bovine) conjugated with horseradish peroxidase (HRP).

The sensitivity and specificity rates reported in the current study are slightly lower compared with those described in a study using ELISA with a *B. bovis* chimerical multi-antigen (rMABbo-ELISA), which achieved sensitivity and specificity values of 95.9% and 94.3%, respectively. Such values may be due to the number of antigenic determinants containing the three *B. bovis* antigens that were used Merozoite Surface Antigen-2c (MSA-2c), Rhoptry-Associated Protein-1 (RAP-1), and heat shock protein 20), which would increase the probability for antibody recognition of *B. bovis* antigens [22]. Another study using more than one antigen (rBbSBP-1 + rBbSBP-4) in an indirect ELISA demonstrated the usefulness of this method for the detection of antibodies against *B. bovis* in cattle compared to results using a single antigen (rBbSBP-1 or rBbSBP-4) [37].

After evaluating the ICT performance in the present study, the specificity rate turned out to be slightly low (83.3%) when compared to that obtained in the evaluation of Bovis ImmunoChromatography Test (BoICT) (93.8%) based on *B. bovis* recombinant MSA-2c antigen [13]. The sensitivity rate of the ICT (90%) is consistent with values reported previously for other developed ICT diagnostic tests for infectious diseases caused by protozoan parasites, with sensitivity rates ranging from 83.3 to 100% [27,38,39,40]. The results presented in our study demonstrate that ICT was more sensitive (90% vs. 87%) and showed greater specificity (83.3% vs. 80%) than ELISA for serological diagnosis. As an explanation for the high number of false positives found in both tests (5/30 and 6/30, respectively) regarding the number of samples, comparing to the values reported for BoICT and Bovis Enzyme-Linked ImmunoSorbent Assay (BoELISA) (5/80 and 4/80, respectively) [13] suggests that the MSA-1 antigen may not be as specific as the MSA-2c antigen for the detection of antibodies against *B. bovis* [41]. It was shown that immunization with a recombinant MSA-1 antigen induced a humoral immune response in cattle [30,42]. Also, specific antibodies against the MSA-1 antigen have been used to assess the in vitro neutralization merozoite invasion of strains of *B. bovis* from Mexico and Texas [43]. Moreover, immunoblotting assays showed cross-reactivity of MSA-1-specific monoclonal antibodies with protein extracts of *B. bovis*-infected erythrocytes from isolates from different regions of Mexico [41]. At present, there is no report on the identification of the orthologous protein of the MSA-1 antigen in protozoan parasites other than *B. bovis*. However, the possibility of cross-reactivity by antibodies against other apicomplexan parasites, such as *N. caninum* and *T. gondii*, for example, cannot be totally dismissed, since this may be the case for the reference bovine serum samples used in the current study, as has been previously described [20,44,45].

The ICT was the most successful test for detecting antibodies against *B. bovis* in field serum samples, showing an overall concordance of 98.6% compared with the IFAT. In a similar study, the concordance of ICT in detecting antibodies against *B. bovis* using the C-terminal truncated portion of the *B. bovis* rhoptry-associated protein 1 (rRAP-1/CTs) was 90.3 and 92.5% compared to the reference standard assays IFAT and ELISA, respectively [26]. The seroprevalence of *B. bovis* by ELISA and ICT (90.3 and 97.2%, respectively) in the current study was higher compared to that documented in other seroepidemiological studies, where the recombinant spherical body protein 4 (SBP-4) of *B. bovis* was used as antigen in the tests (ELISA and ICT). SBP-4 is found in vast quantities in the cytoplasm and is released when merozoites egress from the erythrocyte.

The seroprevalence rates in Uganda using ELISA and ICT based on SBP-4 protein were 6.2 and 4.3%, respectively [14], whereas in Indonesia, *Babesia* prevalence rates were 69.8 and 65.1%, and other studies reported seroprevalence rates of 28.4% and 25.3%, respectively [15,17]. In Argentina, using only the ICT, the determined seroprevalence of *B. bovis* was 71.3% [16]. This notable difference could be because greater numbers of samples were used in those, collected in different regions of the respective countries, as compared to the 72 samples collected in a single region evaluated in the current study, in addition to the difference in breeds of cattle sampled.

When ICT and IFAT results were compared to determine the concordance rate, out of the 72 samples collected at the Pichucalco Experimental Station, 69 were positive in IFAT and 70 were positive in ICT. With these data, the calculated sensitivity and specificity were 100% (69/69) and 66% (2/3), respectively. Possible explanations for the low specificity (66%) of ICT compared to IFAT in terms of concordance, even when serum samples were pre-absorbed with *E. coli* lysates, may be: (a) the presence of a high titer of *E. coli*-reacting antibodies present in the sample, which, despite pre-absorption, remained in the sample, or (b) the presence of cross-reacting antibodies present in samples from cattle previously exposed to, for example, *B. bigemina*, another *Babesia* sp. prevalent in endemic bovine babesiosis areas [25]. This was particularly the case, as the individual was also positive by IFAT when *B. bigemina* infected erythrocytes were used as antigen.

According to the concordance between ELISA and ICT, the highest kappa value calculated in the current study was for ICT (0.79), a similar value to that of bovis ImmunoChromatography Test (*bov*ICT) (>0.7), used to detect antibodies against *B. bovis* in bovine serum samples collected in the field [12,15,17]. The likelihood ratio (LR) is a very useful tool for decision-making when evaluating the performance of a diagnostic test. The interpretation of the LR+ and LR− values indicated that the diagnostic utility of the tests for the serological diagnosis of bovine babesiosis was good for ICT and fair for ELISA according to the scale reported previously by Silva and Molina (2016). In these terms, ICT showed more satisfactory results (5.4 and 0.12, respectively) compared with ELISA (4.3 and 0.16), where LR+ (˃1) and LR− (˂0.1) indicate the diagnostic utility and how appropriate the test might be [29].

## 5. Conclusions

The current study showed that, compared to ELISA, the ICT test showed higher sensitivity, concordance, and diagnostic utility for the detection of antibodies to *B. bovis*. The ICT combines a fast and practical strip-based system, which could be used by technical personnel and veterinarians as a field test for the serodiagnosis of bovine babesiosis in Mexico. The ICT does not require any laboratory equipment, as it can be performed practically on the livestock premises, and the interpretation of test results can be visualized very fast. However, further optimization is recommended to improve the specificity of ICT based on the *B. bovis* rMSA-1 antigen. In order to solve the low specificity of ICT caused by the possible presence of *E. coli* proteins in the test’s gold conjugate, giving rise to potential nonspecific reactions, further purification of recombinant MSA-1 antigen produced in *E. coli* cells should be attempted using other immunoaffinity purification methods with anti-MSA-1-specific antibodies. Better yet, the recombinant protein should be produced in a eukaryotic expression system that releases or secretes it in soluble form in the culture medium, allowing easier protein purification and concentration using, for example, the *Pichia pastoris* expression system. Also, further prevalence studies, including larger numbers of cattle, various cattle production units, and different geographic regions in Mexico, are required to validate the ELISA and ICT.

## Figures and Tables

**Table 1:** Results of The enzyme-linked immunosorbent assay (ELISA), immunochromatography test (ICT), and indirect fluorescent antibody test (IFAT) for detection of antibodies against *B. bovis* in reference serum samples.

Result		Reference Serum Samples	
		*B. bovis*-Positive Serum (n = 30)	*Babesia* spp.-Negative Serum (n = 30)
ELISA	+	26	6
	−	4	24
ICT	+	27	5
	−	3	25
IFAT	+	27	8
	−	3	22

Note: Positive (+) and negative (−) results of each test are shown.

**Table 2:** Results of the ELISA, ICT and IFAT for detection of antibodies to *B. bovis* in field serum samples.

Result ^c^	ELISA		ICT		IFAT	
	n	% ^b^	n ^a^	% ^b^	n ^a^	% ^b^
+	65	90.3	70	97.2	69	95.8
−	7	9.7	2	2.8	3	4.2
Total	72	100	72	100	72	100

^a^ Number of serum samples analyzed. ^b^ Percentage of total number of serum samples analyzed. ^c^ Positive (+) and negative (−) result.

**Table 3:** Comparison of IFAT with ELISA and ICT for detection of antibodies to *B. bovis* in field serum samples.

Result IFAT ^c^	ELISA ^a^		ICT ^b^		Total
	+	−	+	−	
+	65	4	69	0	69
−	0	3	1	2	3
Total	65	7	70	2	72

^a^ Positive (+) and negative (−) result using ELISA test. ^b^ Positive (+) and negative (−) result using ICT test. ^c^ Positive (+) and negative (−) result using IFAT test.

**Table 4:** Overall results of ELISA and ICT for detection of antibodies to *B. bovis* in field serum samples compared with those of IFAT.

Terms Evaluated	IFAT Results Compared to	
	ELISA	ICT
Sensitivity (%)	94.2	100
Specificity (%)	100	66.6
Concordance (%)	94.4	98.6
Kappa value	0.57	0.79
+LR	4.3	5.4
−LR	0.16	0.12

## Data Availability

All data are contained within the paper.
